# Association between medial meniscal extrusion and knee structural progression in adults with symptomatic knee osteoarthritis — a prospective cohort study

**DOI:** 10.1007/s00256-024-04731-2

**Published:** 2024-06-15

**Authors:** Mengjie Zeng, Flavia M. Cicuttini, Anita E. Wluka, Graeme Jones, Catherine L. Hill, Changhai Ding, Yuanyuan Wang

**Affiliations:** 1 School of Public Health and Preventive Medicine, Monash University, 553 St Kilda Road, Melbourne, VIC 3004 Australia; 2 Menzies Institute for Medical Research, University of Tasmania, Hobart, TAS 7000 Australia; 3 The Queen Elizabeth Hospital, University of Adelaide, Woodville, SA 5011 Australia; 4 Department of Medicine, University of Adelaide, Adelaide, SA 5000 Australia; 5 Clinical Research Centre, Zhujiang Hospital, Southern Medical University, Guangdong, China

**Keywords:** Meniscal extrusion, Knee osteoarthritis, Bone marrow lesions, Magnetic resonance imaging

## Abstract

**Objective:**

To examine the association between medial meniscal extrusion and structural progression in adults with symptomatic knee osteoarthritis (OA).

**Methods:**

This prospective cohort study examined 176 participants with symptomatic knee OA recruited into a randomised controlled trial. The participants underwent magnetic resonance imaging (MRI) of the study knee at baseline and approximately 2 years later. Meniscal extrusion, tibial cartilage volume, and tibiofemoral bone marrow lesions (BMLs) were measured from MRI using validated methods.

**Results:**

Participants with medial meniscal extrusion ≥ 3 mm had a higher prevalence of lateral tibiofemoral BMLs at baseline (OR = 2.21, 95% CI 1.06–4.61, *p* = 0.035), and those with medial meniscal extrusion 2–3 mm had a higher likelihood of lateral BML worsening over 2 years (OR = 3.76, 95% CI 1.35–10.52, *p* = 0.011), compared with those with medial meniscal extrusion < 2 mm. Participants with stable medial meniscal extrusion had a lower likelihood of lateral BML worsening compared with those with regression of medial meniscal extrusion over 2 years (OR = 0.20, 95% CI 0.07–0.56, *p* = 0.002). There were no associations between medial meniscal extrusion and tibial cartilage volume or medial tibiofemoral BMLs.

**Conclusions:**

Our study showed associations between medial meniscal extrusion and baseline prevalence and worsening over 2 years of lateral tibiofemoral BMLs in people with symptomatic knee OA. Although the reasons for the lack of associations in the medial compartment are not clear, our results suggest a role of medial meniscal extrusion in predicting structural progression in lateral knee OA and that meniscal extrusion might be a potential target in the management of knee OA.

## Introduction

Knee osteoarthritis (OA) is the most common chronic joint disease, affecting over 300 million people worldwide [1]. It affects the whole joint and is characterised by progressive cartilage loss accompanied by subchondral bone modelling, synovial proliferation, and deterioration of tendons and ligaments. These structural changes result in pain, stiffness, loss of function, and decreased mobility [2]. In knee OA, the meniscus can undergo degenerative changes, including loss of volume and extrusion.

The medial meniscus is a crescent-shaped fibrocartilage structure which plays a key role in shock absorption, distributing mechanical loads on the articular cartilage and stabilising the knee joint [3]. Meniscal extrusion refers to the displacement of the meniscus beyond the edge of the tibial plateau from its normal position within the knee joint [4], being either partially or totally displaced from the tibial cartilage surface [5]. It is a common finding in individuals with knee OA and has been associated with an increased risk of structural progression in knee OA, evidenced by accelerated cartilage loss and increased bone marrow lesions (BMLs) [6, 7]. The general idea is that a displaced meniscus affects the weight-bearing and load distribution within the knee joint, which results in damage to articular cartilage and subchondral bone, ultimately leading to the development and progression of knee OA [8–10]. Previous studies have confirmed the relationship between medial meniscal extrusion and cartilage loss and BMLs in the ipsilateral compartment [9–11]. There is evidence from biomechanical analysis that damage of the medial meniscus leads to high deformation and stress on the lateral meniscus, therefore increasing stress application on the cartilages and bone structures [12]. However, no previous studies have investigated whether there is a link between medial meniscal extrusion and cartilage damage and BMLs in the lateral tibiofemoral compartment.

Therefore, the aim of our study was to comprehensively investigate the associations of medial meniscal extrusion and its changes with knee structural progression over 2 years in the medial and lateral compartment separately in a cohort of adults with symptomatic knee OA. It was hypothesised that the presence of medial meniscal extrusion at baseline and its progression over 2 years would be associated with knee structural progression in both medial and lateral compartments.

## Methods

### Study participants

**Fig. 1:**
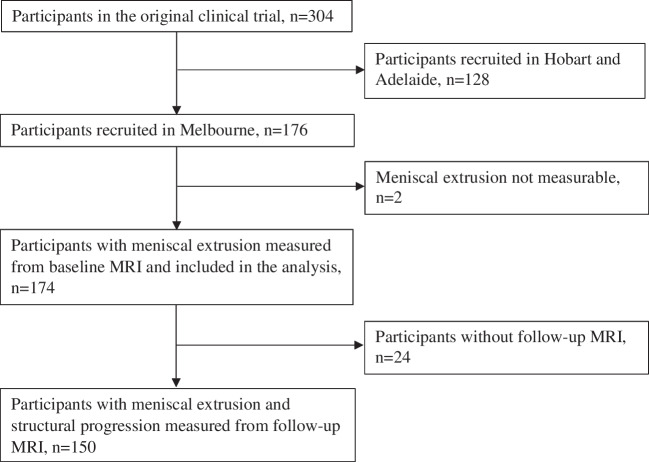
Flow diagram of study participants

The osteoarthritis of the knee statin (OAKS) study was a randomised controlled trial examining the effect of atorvastatin on the progression of knee OA, with 304 participants recruited in Melbourne, Hobart, and Adelaide, Australia [13, 14]. Eligible participants were aged 40–70 years with symptomatic knee OA which fulfilled the American College of Rheumatology clinical criteria [15] and with a pain score of ≥ 20 mm on a 100-mm visual analogue scale. Participants were excluded if they had severe radiographic knee OA (grade 3 joint space narrowing according to Altman’s atlas [16]); severe knee pain; inflammatory arthritis; significant knee injury; accepted indications for statin therapy; current use of lipid-lowering therapy, or previous adverse reaction to statins; absolute cardiovascular risk estimated using the Framingham risk equation of > 15% within the next 5 years; fasting total cholesterol level > 7.5 mmol/L; clinically significant renal disease or abnormal liver function; arthroscopy or open surgery or intra-articular therapy in the index knee in the last 12 months; concomitant use of potent analgesics including opiates; comorbidity limiting participation; relocation; contraindication to magnetic resonance imaging (MRI) scanning; pregnancy, breastfeeding, or women trying to become pregnant, or inability to give informed consent [13, 14]. The trial was registered with the Australian New Zealand Clinical Trials Registry (ACTRN12613000190707). Ethics approval was obtained from the Alfred Hospital Ethics Committee, Monash University Human Research Ethics Committee, Tasmania Health and Medical Human Research Ethics Committee, and The Queen Elizabeth Hospital Human Research Ethics Committee. Written informed consent was obtained from all the participants. This current study included 176 participants recruited in Melbourne who had coronal MRI performed for meniscal extrusion measurement. Among these participants, 152 underwent a follow-up MRI approximately two years later (Fig. 1).

### Knee MRI acquisition

MRI was conducted on the study knee using a 3.0 T whole-body MR unit utilising a commercial 16-channel transmit-receive knee coil (Achieva, Philips Medical Systems). T1-weighted fat-suppressed 3D gradient recall acquisition in a steady state was employed for the measurement of cartilage volume [14]. Proton density fat-saturated acquisition was used to assess bone marrow lesions (sagittal) and meniscal extrusion (coronal) [14]. Details of MRI units, sequences, and parameters have been published previously [14].

### Assessment of medial meniscal extrusion

**Fig. 2:**
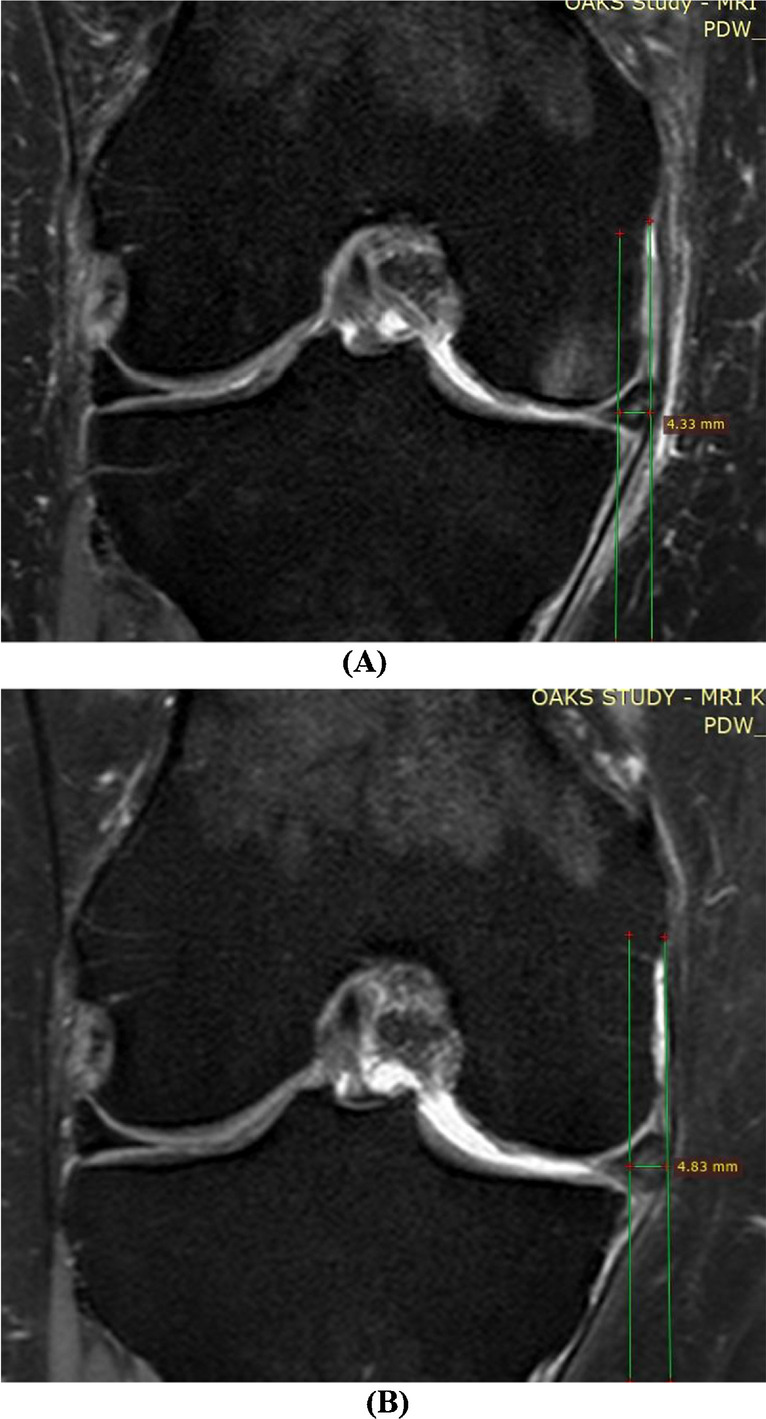
Measurement of medial meniscal extrusion. **A** Baseline MRI; **B** follow-up MRI of the same participant. Medial meniscal extrusion was measured on the coronal plane at the slice with the largest area of the medial tibial spine with the steps: (1) drop a perpendicular line at the point of medial tibial plateau transitioning from horizontal to vertical; (2) drop another perpendicular line intersecting the outermost edge of the medial meniscus; (3) measure meniscal extrusion as the distance between the two perpendicular lines

Medial meniscal extrusion was defined as the displacement of the medial meniscus beyond the innermost border of the tibial plateau, as measured through the knee joint in the coronal plane [17]. The coronal plane for meniscal extrusion measurement was defined as the slice with the largest area of the medial tibial spine. If the maximum area of the medial spine could not be distinguished on two consecutive slices, the slice with the widest tibial plateau was selected [18]. If there were bone spurs present, they were excluded from the measurement [19]. Extrusion of the medial meniscal body was assessed using validated methods [20] using the software RadiAnt DICOM Viewer (Fig. 2). The steps of measurement include (1) dropping a perpendicular line at the point of medial tibial plateau transitioning from horizontal to vertical, (2) dropping another perpendicular line intersecting the outermost edge of the medial meniscus, and (3) measuring meniscal extrusion as the distance between the two perpendicular lines in mm. One trained observer performed the measurement in duplicate, with an intra-observer reproducibility (intra-class correlation coefficient) of 0.99, and the average results were taken as the final measure. The grade of medial meniscal extrusion was defined as 0–2: grade 0: extrusion < 2 mm; grade 1: extrusion 2–3 mm; and grade 2: extrusion ≥ 3 mm [6, 21]. The change in medial meniscal extrusion over 2 years was defined as follows: regression (baseline extrusion grade > follow-up extrusion grade), stable (baseline extrusion grade = follow-up extrusion grade), and progression (baseline extrusion grade < follow-up extrusion grade).

### Measurement of tibial cartilage volume

Medial and lateral tibial cartilage volume was measured from sagittal T1-weighted images by manually drawing the disarticulation contours around the cartilage boundaries on a section-by-section basis, using the OsiriX software. Two trained observers independently performed the measurement with an inter-observer intraclass correlation coefficient of 0.91, and the average results were taken as the final measure. The annual percentage change in medial and lateral tibial cartilage volume was calculated separately as (follow-up cartilage volume − baseline cartilage volume)/baseline cartilage volume/years between MRI scans*100 [13].

### Assessment of bone marrow lesions

**Fig. 3:**
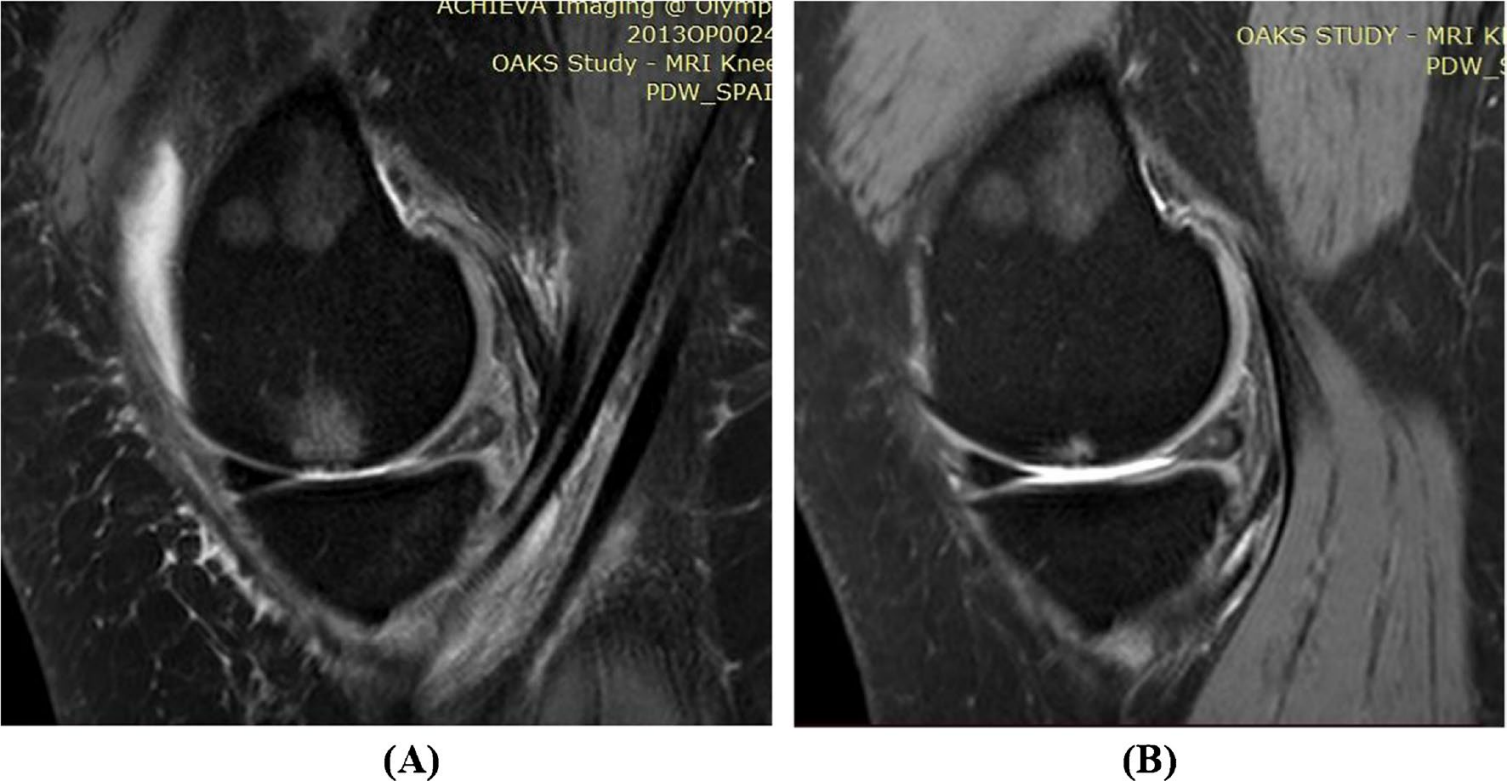
Bone marrow lesions at baseline and follow-up. **A** Baseline MRI, showing a grade 3 bone marrow lesion in the medial femur; **B** follow-up MRI of the same participant, showing a grade 1 bone marrow lesion in the same subregion

BMLs were defined as areas of increased signal intensity adjacent to the subcortical bone in either the distal femur or the proximal tibia [22]. A lesion was identified as being present if it appeared on two or more adjacent slices. BMLs were graded at medial and lateral tibial and femoral sites (0–3) from sagittal proton density images using the MOAKS [23], where grade 0, none; grade 1, < 33% of subregion; grade 2, 33–66% of subregion; grade 3, > 66% of subregion (Fig. 3). One trained observer performed the measurement, with random cross-check performed by a second observer independently, with intra- and inter-reader intraclass correlation coefficients of 0.88–0.93 [13]. The discrepancy in grading was solved by a discussion between the two observers. The prevalence of BMLs was defined as a grade ≥ 1 in either the tibial or femoral site in the medial and lateral compartments. The worsening of BMLs was defined as any increase in grade in either the tibial site or femoral site over 2 years in the medial and lateral compartments.

### Tibial bone area measurement

The cross-sectional areas of the medial and lateral tibial plateau were measured from axial images as previously described [24]. The coefficients of variation for the medial and lateral tibial plateau areas were 2.3% and 2.4%, respectively [24].

### Anthropometric measurements

At baseline, height (stadiometer) and weight (electric scale) were measured, and body mass index (BMI, height/weight^2^) was calculated.

### Statistical analyses

With 176 participants, the current study had 80% power to detect a correlation coefficient as low as 0.21 between meniscal extrusion and knee structural progression, alpha 0.05, two-sided significance. Participant characteristics were tabulated and compared among participants with grade 0, 1, and 2 medial meniscal extrusion using analysis of variance (ANOVA) and chi-square test, when appropriate. The differences in medial and lateral tibial cartilage volume at baseline or annual percentage change in medial and lateral tibial cartilage volume among participants with grade 0, 1, and 2 medial meniscal extrusion were examined using *F*-test (general linear model) with estimated marginal means. The analyses were adjusted for age, sex, BMI, and baseline tibial plateau bone area, with the analysis of annual percentage change in cartilage volume additionally adjusted for randomisation group allocation (atorvastatin/placebo). The associations between medial meniscal extrusion and the prevalence of medial and lateral tibiofemoral BMLs were examined using binary logistic regression, adjusting for age, sex, and BMI. The associations between medial meniscal extrusion and worsening of medial and lateral tibiofemoral BMLs were also examined using binary logistic regression, adjusting for age, sex, BMI, randomisation group allocation, and baseline BML score. Given the association between lateral meniscal lesion and BMLs in the lateral compartment [9], additional adjustment for lateral meniscal extrusion was performed in the analysis of the association between medial meniscal extrusion and BMLs in the lateral compartment. *P*-values of less than 0.05 were considered statistically significant. All analyses were performed using the SPSS statistical package (standard version 28, SPSS, USA).

## Results

Characteristics of study participants are presented in Table 1. Medial meniscal extrusion was measured in 174 participants, but not measurable for 2 participants as no coronal images (Fig. 1). The current study included 174 participants, 81 (46.5%) with grade 0 meniscal extrusion, 41 (23.6%) with grade 1 and 52 (29.9%) with grade 2 meniscal extrusion. The prevalence of lateral tibiofemoral BMLs was different among the three groups, which was higher in participants with grade 2 medical extrusion compared with the other two groups. There were no significant differences among the three groups in other characteristics.

**Table 1:** Characteristics of study participants at baseline

Characteristics	Grade 0 meniscal extrusion (< 2 mm) *N* = 81	Grade 1 meniscal extrusion (2–3 mm) *N* = 41	Grade 2 meniscal extrusion (≥ 3 mm) *N* = 52	p^*^
Age, years	56.0 (7.5)	55.0 (5.9)	58.0 (7.3)	0.109
Female, *n* (%)	45 (55.6)	24 (58.5)	31 (59.6)	0.888
Body mass index, kg/m^2^	28.7 (5.6)	29.2 (4.9)	30.7 (5.8)	0.164
Joint space narrowing^¶^, no (%)				0.159
Grade 0	38 (50.0)	18 (45.0)	18 (35.3)	
Grade 1	23 (30.3)	13 (32.5)	13 (25.5)	
Grade 2	15 (19.7)	9 (22.5)	20 (39.2)	
Atorvastatin, no (%)	41 (50.6)	17 (41.5)	30 (57.7)	0.297
Medial tibial cartilage volume, mm^3^	1712.0 (496.9)	1767.5 (546.3)	1855.1 (537.0)	0.305
Lateral tibial cartilage volume, mm^3^	1966.6 (676.2)	1889.4 (651.3)	1927.2 (544.2)	0.810
Medial tibial bone area, mm^2^	22.4 (4.0)	22.4 (4.0)	23.4 (3.9)	0.284
Lateral tibial bone area, mm^2^	13.0 (2.8)	12.8 (2.5)	13.6 (2.9)	0.278
Prevalence of medial tibiofemoral bone marrow lesions, *n* (%)	29 (35.8)	20 (48.8)	26 (50.0)	0.191
Prevalence of lateral tibiofemoral bone marrow lesions, *n* (%)	27 (33.3)	13 (31.7)	28 (53.8)	0.033

Data presented as mean (standard deviation) or *n* (%)

^*^Significance for differences among three groups using analysis of variance (ANOVA) or chi-square tests

^¶^Data missing for seven participants

The associations between medial meniscal extrusion and knee structure at baseline are summarized in Table 2. There were no significant associations between medial meniscal extrusion grade and medial tibial cartilage volume or the prevalence of medial tibiofemoral BMLs in univariable or multivariable analyses. The univariable analysis showed a higher prevalence of lateral tibiofemoral BMLs in participants with grade 2 medial meniscal extrusion compared with those with grade 0 medial meniscal extrusion, which persisted in multivariable analysis adjusted for age, sex, and BMI (odds ratio (OR) 2.21, 95% confidence interval (CI) 1.06–4.61), or with additional adjustment for lateral meniscal extrusion (OR 2.26, 95% CI 1.08–4.74, *p* = 0.031). There was no significant association between medial meniscal extrusion grade and lateral tibial cartilage volume in univariable or multivariable analysis.

**Table 2:** Association between medial meniscal extrusion and knee structure at baseline

	Univariable analysis		Multivariable analysis	
	*Medial tibial cartilage volume* *Mean (SD)*	*p*	*Medial tibial cartilage volume*^*a*^ *Mean (SE)*	*p*
Extrusion grade 0	1711.9 (57.9)	0.305	1724.8 (42.8)	0.336
Extrusion grade 1	1767.5 (81.4)		1777.4 (60.2)	
Extrusion grade 2	1855.1 (72.2)		1827.3 (54.4)	
	*Prevalence of medial tibiofemoral BMLs* *OR (95% CI)*	*p*	*Prevalence of medial tibiofemoral BMLs*^*b*^ *OR (95% CI)*	*p*
Extrusion grade 0	1.00		1.00	
Extrusion grade 1	1.71 (0.80, 3.66)	0.169	1.81 (0.83, 3.99)	0.138
Extrusion grade 2	1.79 (0.88, 3.64)	0.106	1.62 (0.78, 3.40)	0.198
	*Lateral tibial cartilage volume* *Mean (SD)*	*p*	*Lateral tibial cartilage volume*^*a*^ *Mean (SE)*	*p*
Extrusion grade 0	1966.6 (70.4)	0.810	1952.1 (53.1)	0.762
Extrusion grade 1	1889.4 (98.9)		1888.3 (74.9)	
Extrusion grade 2	1927.2 (87.9)		1950.7 (67.5)	
	*Prevalence of lateral tibiofemoral BMLs* *OR (95% CI)*	*p*	*Prevalence of lateral tibiofemoral BMLs*^*b*^ *OR (95% CI)*	*p*
Extrusion grade 0	1.00		1.00	
Extrusion grade 1	0.93 (0.42, 2.07)	0.857	0.98 (0.43, 2.22)	0.964
Extrusion grade 2	2.33 (1.14, 4.77)	0.020	2.21 (1.06, 4.61)	0.035

^a^Adjusted for age, sex, BMI, and tibial plateau bone area

^b^Adjusted for age, sex, and BMI

The associations between medial meniscal extrusion at baseline and changes in knee structure over 2 years are presented in Table 3. Worsening of BMLs was observed in 66/150 (44.0%) participants for the medial compartment and 34/150 (22.7%) participants for the lateral compartment. There was no significant association between medial meniscal extrusion grade and annual percentage change in medial tibial cartilage volume, annual percentage change in lateral tibial cartilage volume, or medial BML worsening, in either univariable or multivariable analyses. In univariable analysis, there was a higher likelihood of lateral BML worsening in participants with grade 1 medial meniscal extrusion compared with those with grade 0 medial meniscal extrusion, which persisted in multivariable analysis adjusted for age, sex, BMI, randomisation group allocation, and baseline BML score (OR 3.76, 95% CI 1.35–10.52), or with additional adjustment for lateral meniscal extrusion (OR 3.84, 95% CI 1.37–10.80, *p* = 0.011).

**Table 3:** Association between medial meniscal extrusion at baseline and changes in knee structure over 2 years

	Univariable analysis		Multivariable analysis	
	*Annual % change in medial tibial cartilage volume* *Mean (SD)*	*p*	*Annual % change medial tibial cartilage volume*^*a*^ *Mean (SE)*	*p*
Extrusion grade 0	− 0.94 (0.38)	0.747	− 0.99 (0.39)	0.849
Extrusion grade 1	− 1.42 (0.54)		− 1.38 (0.55)	
Extrusion grade 2	− 1.22 (0.45)		− 1.17 (0.47)	
	*Worsening of medial BMLs* *OR (95% CI)*	*p*	*Worsening of medial BMLs*^*b*^ *OR (95% CI)*	*p*
Extrusion grade 0	1.00		1.00	
Extrusion grade 1	1.20 (0.52, 2.76)	0.670	1.03 (0.42, 2.52)	0.944
Extrusion grade 2	1.52 (0.72, 3.20)	0.272	1.16 (0.52, 2.61)	0.720
	*Annual % change in lateral tibial cartilage volume* *Mean (SD)*	*p*	*Annual % change in lateral tibial cartilage volume*^*a*^ *Mean (SE)*	*p*
Extrusion grade 0	− 1.74 (0.29)	0.973	− 1.78 (0.29)	0.979
Extrusion grade 1	− 1.75 (0.41)		− 1.70 (0.42)	
Extrusion grade 2	− 1.84 (0.35)		− 1.82 (0.36)	
	*Worsening of lateral BMLs* *OR (95% CI)*	*p*	*Worsening of lateral BMLs*^*b*^ *OR (95% CI)*	*p*
Extrusion grade 0	1.00		1.00	
Extrusion grade 1	3.57 (1.32, 9.66)	0.012	3.76 (1.35, 10.52)	0.011
Extrusion grade 2	2.44 (0.94, 6.28)	0.066	2.13 (0.80, 5.66)	0.130

^a^Adjusted for age, sex, BMI, randomisation group allocation, and baseline tibial plateau bone area

^b^Adjusted for age, sex, BMI, randomisation group allocation, and baseline BML score

In terms of the change in medial meniscal extrusion, regression was observed in 14.2% of the participants, stable extrusion in 55.1%, and progression in 14.2% of the participants. The associations between changes in medial meniscal extrusion grade and changes in knee structure over 2 years are shown in Table 4. There was no significant association between change in medial meniscal extrusion grade and annual percentage change in medial tibial cartilage volume, annual percentage change in lateral tibial cartilage volume, or medial BML worsening, in either univariable or multivariable analyses. In the univariable analysis, there was a lower likelihood of lateral BML worsening in participants with stable medial meniscal extrusion compared with those with regressed medial meniscal extrusion. The significant association persisted in multivariable analysis adjusted for age, sex, BMI, randomisation group allocation, and baseline BML score (OR 0.20, 95% CI 0.07–0.56), or with additional adjustment for lateral meniscal extrusion (OR 0.15, 95% CI 0.05–0.47, *p* = 0.001).

**Table 4:** Association between change in medial meniscal extrusion grade and changes in knee structure over 2 years

	Univariable analysis		Multivariable analysis	
	*Annual % change in medial tibial cartilage volume* *Mean (SD)*	*p*	*Annual % change in medial tibial cartilage volume*^*a*^ *Mean (SE)*	*p*
Regression of extrusion	− 1.46 (0.62)	0.645	− 1.44 (0.64)	0.641
Stable extrusion	− 0.87 (0.31)		− 0.86 (0.32)	
Progression of extrusion	− 1.26 (0.62)		− 1.33 (0.63)	
	*Worsening of medial BMLs* *OR (95% CI)*	*p*	*Worsening of medial BMLs*^*b*^ *OR (95% CI)*	*p*
Regression of extrusion	1.00		1.00	
Stable extrusion	1.67 (0.67, 4.15)	0.268	1.73 (0.64, 4.74)	0.283
Progression of extrusion	0.84 (0.26, 2.70)	0.765	0.93 (0.26, 3.34)	0.908
	*Annual % change in lateral tibial cartilage volume* *Mean (SD)*	*p*	*Annual % change in lateral tibial cartilage volume*^*a*^ *Mean (SE)*	*p*
Regression of extrusion	− 2.59 (0.48)	0.193	− 2.60 (0.49)	0.200
Stable extrusion	− 1.63 (0.24)		− 1.62 (0.25)	
Progression of extrusion	− 1.65 (0.48)		− 1.65 (0.48)	
	*Worsening of lateral BMLs* *OR (95% CI)*	*p*	*Worsening of lateral BMLs*^*b*^ *OR (95% CI)*	*p*
Regression of extrusion	1.00		1.00	
Stable extrusion	0.27 (0.11, 0.70)	0.007	0.20 (0.07, 0.56)	0.002
Progression of extrusion	0.40 (0.12, 1.35)	0.140	0.31 (0.08, 1.16)	0.081

^a^Adjusted for age, sex, BMI, randomisation group allocation, and baseline tibial plateau bone area

^b^Adjusted for age, sex, BMI, randomisation group allocation, and baseline BML score

## Discussion

Our prospective cohort study showed associations between medial meniscal extrusion and BMLs in the lateral tibiofemoral compartment in individuals with symptomatic knee OA. Cross-sectionally, participants with severe medial meniscal extrusion (grade 2, ≥ 3 mm) had a higher prevalence of lateral tibiofemoral BMLs compared with those with grade 0 extrusion (< 2 mm) at baseline. Longitudinally, a higher likelihood of lateral BML worsening over 2 years was observed in participants with grade 1 medial meniscal extrusion (2–3 mm) compared with those with grade 0 extrusion at baseline. Moreover, participants with stable medial meniscal extrusion over 2 years had a lower likelihood of lateral BML worsening over 2 years compared with those with regressed medial meniscal extrusion.

The associations between medial meniscal extrusion and the development and progression of knee OA, as well as structural progression in the medial compartment, have been examined, showing consistent results [9–11]. In the current study, we found associations between baseline medial meniscal extrusion and the prevalence and worsening of BMLs in the lateral tibiofemoral compartment, with no significant associations observed for BMLs in the medial compartment. BMLs are commonly associated with knee OA, being associated with knee pain and cartilage loss, and predictive of total knee replacement [25–27]. BMLs are affected by joint loading and the response of joint tissues to this abnormal biomechanical stress, as well as systematic factors [28, 29]. BML is not a static phenomenon but changes over time, and the natural history of BMLs varies in individuals with knee OA [30–32]. In our study, worsening of BMLs in the medial and lateral compartment over 2 years was observed in 44.0% and 22.7% of participants, respectively. An increase in BML size has been shown to be associated with increased knee pain and progressive cartilage loss [33, 34]. Previous studies have shown the relationship between medial meniscal extrusion and BMLs in the medial tibiofemoral compartment [9, 10] with no studies examining whether there is a link between medial meniscal extrusion and lateral BMLs. Biomechanical analysis has demonstrated increased deformation and stress on the lateral meniscus as a result of damage to the medial meniscus, which increased the stress on the cartilage and bone of the lateral compartment [12]. It is biologically plausible that medial meniscal extrusion would lead to structural damage in the lateral tibiofemoral compartment. To our knowledge, our study is the first to report consistent associations between medial meniscal extrusion and the prevalence and worsening of lateral tibiofemoral BMLs in both cross-sectional and longitudinal analyses. Our study also adds to the existing literature by exploring the association between changes in medial meniscal extrusion and changes in knee structural outcomes over 2 years, showing an increased likelihood of lateral BML worsening in participants with regressed medial meniscal extrusion compared with those with stable meniscal extrusion. The potential mechanism behind this link is unknown but may relate to the altered biomechanics and increased contact pressure on the lateral tibiofemoral joint surfaces attributable to the severity and/or change in medial meniscal extrusion, which may cause BMLs. Compared with stable medial meniscal extrusion over 2 years, regression in medial meniscal extrusion may cause varus thrust with tibial spine impingement on the lateral femoral condyle under axial load, which might change the biomechanics of the lateral compartment and provide a potential explanation for the increased likelihood of BML worsening in that compartment. The relationship between meniscal extrusion and structural progression in the contralateral compartment warrants further investigation.

In contrast to previous studies [7, 11], our study found no association between medial meniscal extrusion and medial tibial cartilage volume or medial tibiofemoral BMLs. This might be due to different study populations or different methods for assessment of meniscal extrusion. It might suggest that the potential mechanism behind these structural changes in knee OA may not be directly related to medial meniscal extrusion. It may be that the effect of meniscal extrusion is less apparent in more severe OA. As participants in this study tended to have more severe OA in the medial than the lateral compartment, it may be that other factors including other biomechanical factors play a more significant role in the structural progression of the medial compartment. Further research is needed to better understand the complex interplay of biomechanical and systemic factors that contribute to the structural progression of knee OA.

There are some limitations that should be considered. Firstly, meniscal extrusion might be underestimated on supine MRI scans [35], which may not accurately reflect the prevalence of meniscal extrusion during usual activities. Most of the participants in our study had mild to moderate knee OA [13], so the findings may not be generalisable to healthy individuals without knee OA or patients with severe disease. We examined changes in meniscal extrusion, cartilage volume, and BMLs over a 2-year period. It is unclear whether changes in meniscal extrusion cause changes in cartilage and BMLs, or vice versa, which limits the ability to establish causality. The relationship between these variables is complex and requires further investigation to establish causality. Femorotibial alignment, which might be a covariate for the association between meniscal extrusion and structural progression, in particular BMLs, was not measured in the current study. Meniscal root tear, which is associated with meniscal extrusion and structural progression in knee OA, was not measured in our study. Whether the association between meniscal extrusion and structural progression is mediated by meniscal tears warrants further investigation. Further studies of larger sample size and/or with longer follow-up are needed to better understand the relationship between meniscal extrusion and structural progression in knee OA. The strength of the study was the investigation of association between medial meniscal extrusion and structural progression in the medial and lateral tibiofemoral compartment separately and the examination of changes in meniscal extrusion over 2 years. There were consistent associations between medial meniscal extrusion and lateral tibiofemoral BMLs in both cross-sectional and longitudinal analyses, strengthening our findings. Since knee OA is a progressive disease, understanding the role of modifiable factors, such as meniscal extrusion, in structural progression may have the potential to inform therapeutic strategies.

## Conclusions

Our study demonstrated associations between medial meniscal extrusion and baseline prevalence and worsening over 2 years of BMLs in the lateral tibiofemoral compartment in individuals with symptomatic knee OA. The results suggest that medial meniscal extrusion may be a predictor of structural progression in knee OA, in particular worsening of BMLs in the lateral tibiofemoral compartment. These findings have implications for better understanding the pathogenesis of knee OA and identifying potential targets for disease prevention and treatment.

## Data Availability

The data generated from this study will not be deposited in a public repository due to privacy and consent restrictions. Deidentified data can be made available from the corresponding author on reasonable request, subject to a data-sharing agreement.
